# Factors associated with dose reduction of pirfenidone in patients with idiopathic pulmonary fibrosis: A study based on real-world clinical data

**DOI:** 10.1371/journal.pone.0281295

**Published:** 2023-02-03

**Authors:** Jiwon Kim, Chiwook Chung, Hyo Sin Cho, Ho Cheol Kim

**Affiliations:** 1 Department of Pulmonary and Critical Care Medicine, University of Ulsan College of Medicine, Asan Medical Center, Seoul, Republic of Korea; 2 University of Ulsan College of Medicine, Seoul, Republic of Korea; University of Miami School of Medicine, UNITED STATES

## Abstract

**Introduction:**

Although pirfenidone slows disease progression in patients with idiopathic pulmonary fibrosis (IPF), in clinical practice, patients often cannot tolerate the recommended dose because of several adverse events. This study aimed to investigate adverse events associated with pirfenidone and factors associated with dose reduction.

**Methods:**

This single-center retrospective cohort study included 156 consecutive patients with IPF who received pirfenidone. Demographic characteristics, pulmonary function, and pirfenidone-related adverse events were investigated. We compared patients who received standard and reduced doses of pirfenidone.

**Results:**

The mean patient age was 69.7 years. The median follow-up duration was 243 days. The low-dose group (n = 73) included older patients (71.0 years vs. 67.4 years, *p* = 0.016), fewer smokers (80.8% vs. 96.4%, *p* = 0.008), and patients with a lower body mass index (BMI; 24.1 kg/m^2^ vs. 25.7 kg/m^2^, *p* = 0.027) than the standard dose group (n = 57). Multivariate logistic regression analysis revealed that older age (odds ratio = 1.066, *p* = 0.016) was significantly associated with dose reduction of pirfenidone after adjusting for sex, smoking history, emphysema, and BMI. No significant difference was found in the rates of a reduced forced vital capacity and diffusing capacity for carbon monoxide between the two groups.

**Conclusions:**

Although older patients are more likely to undergo dose reduction of pirfenidone, low-dose pirfenidone might be effective for treating patients with IPF. Low-dose pirfenidone could be considered an effective treatment option for older patients with IPF.

## Introduction

Idiopathic pulmonary fibrosis (IPF) is a chronic, progressive, and fibrotic interstitial lung disease [1]. The clinical course of IPF is highly variable and unpredictable, with a median survival of 3–5 years after diagnosis [2, 3].

The antifibrotic drug, pirfenidone, reduces lung function decline in patients with IPF compared with placebo [4, 5] and is used in clinical practice [6, 7]. In addition, several recent studies have reported that pirfenidone might reduce mortality in patients with IPF [8, 9].

However, it is well known that pirfenidone may cause side effects. Gastrointestinal and skin-related events are common side effects of pirfenidone [4, 5, 10, 11]. In a clinical trial conducted in Japan, the efficacy of 1800 mg/day of pirfenidone was evaluated, which was lower than the dose administered (2403 mg/day) in the United States, Europe, Australia, and North America. However, the dose of 1800 mg/day was thought to be comparable to 2403 mg/day on a weight-normalized basis [5, 12]. According to a recent post-marketing surveillance (PMS) study conducted in Korea, 86.3% of patients experienced adverse events [13], suggesting that the number of side effects of pirfenidone is likely to be higher in Asia, especially in real-world practice than in randomized controlled trials (RCTs).

Although some studies have reported that low-dose pirfenidone is not inferior to a standard dose [12, 14, 15], there are few reports focused on the side effects associated with pirfenidone and reduced doses. Thus, we aimed to investigate clinical characteristics of and factors associated with dose reduction of pirfenidone in a real-world setting.

## Materials and methods

### Patients and settings

This single-center retrospective cohort study enrolled patients with IPF from the Asan Medical Center, Seoul, Republic of Korea. Patients who started pirfenidone therapy between August 2017 and July 2021 were identified using electronic medical records (EMRs).

The inclusion criteria were as follows: (1) diagnosis of IPF, (2) treatment with pirfenidone, and (3) at least one follow-up visit after pirfenidone administration to assess the occurrence of adverse events. IPF was diagnosed based on the American Thoracic Society/European Respiratory Society/Japanese Respiratory Society/Latin American Thoracic Association guidelines published in 2018 [1].

Demographic and clinical data, including age, sex, body mass index (BMI), smoking status, comorbidities, concurrent medication usage, pulmonary function test (PFT) results, six-minute walk test (6MWT) results, high-resolution computed tomography (HRCT), echocardiogram findings, prescribing information for pirfenidone therapy, reasons for dose reduction, and adverse events, were collected from EMRs. Spirometry was performed, and the diffusing capacity of the lungs for carbon monoxide (DLCO) was measured. Pulmonary hypertension was diagnosed based on echocardiographic evidence: maximal tricuspid regurgitation velocity (TR Vmax) >3.4 m/s or estimated systolic pulmonary arterial pressure >40 mmHg [16, 17].

The study was conducted in accordance with the tenets of the Declaration of Helsinki. The study protocol was approved by the Institutional Review Board of Asan Medical Center (IRB number: 2021–0787). The requirement for informed consent was waived because of the retrospective nature of the study and use of anonymized clinical data for analysis.

### Pirfenidone dose

In Korea and Japan, 1800 mg of pirfenidone has been used as the standard dose, which is thought to be comparable to 2403 mg/day on a weight-normalized basis [5, 12, 13]. In our study, patients diagnosed with IPF were initially prescribed 600 mg/day of pirfenidone. The dose was increased every 1–2 weeks until it reached 1800 mg/day. The patients were divided into the following two groups: those treated with a standard dose of pirfenidone (1800 mg/day) and those treated with low-dose pirfenidone (<1800 mg/day). The standard dose group included patients who continued taking the standard dose of pirfenidone (1800 mg/day) after dose escalation. The low-dose group included patients who did not receive higher doses of the drug (maximum: standard dose [1800 mg/day]) and those who required reduced doses of the drug.

### Statistical analysis

The baseline characteristics of all patients are presented as numbers (percentages; categorical variables) or as the mean ± standard deviation (continuous variables). Continuous and categorical variables were compared between the groups using an independent t test and Fisher’s exact test or Pearson’s chi-square test, respectively. Receiver operating characteristic (ROC) curve analysis was used to identify the optimal cutoff value. Univariate and multivariate logistic regression analyses were performed to evaluate the factors associated with dose reduction of pirfenidone.

In addition, a linear mixed model was used to compare the treatment effect according to the dose of pirfenidone. Forced vital capacity (FVC) and DLCO data of patients who underwent at least two PFTs after receiving pirfenidone were used for analysis.

Statistical analysis was performed using SPSS version 26. A *p* value of <0.05 was considered statistically significant.

## Results

### Characteristics

**Fig 1:**
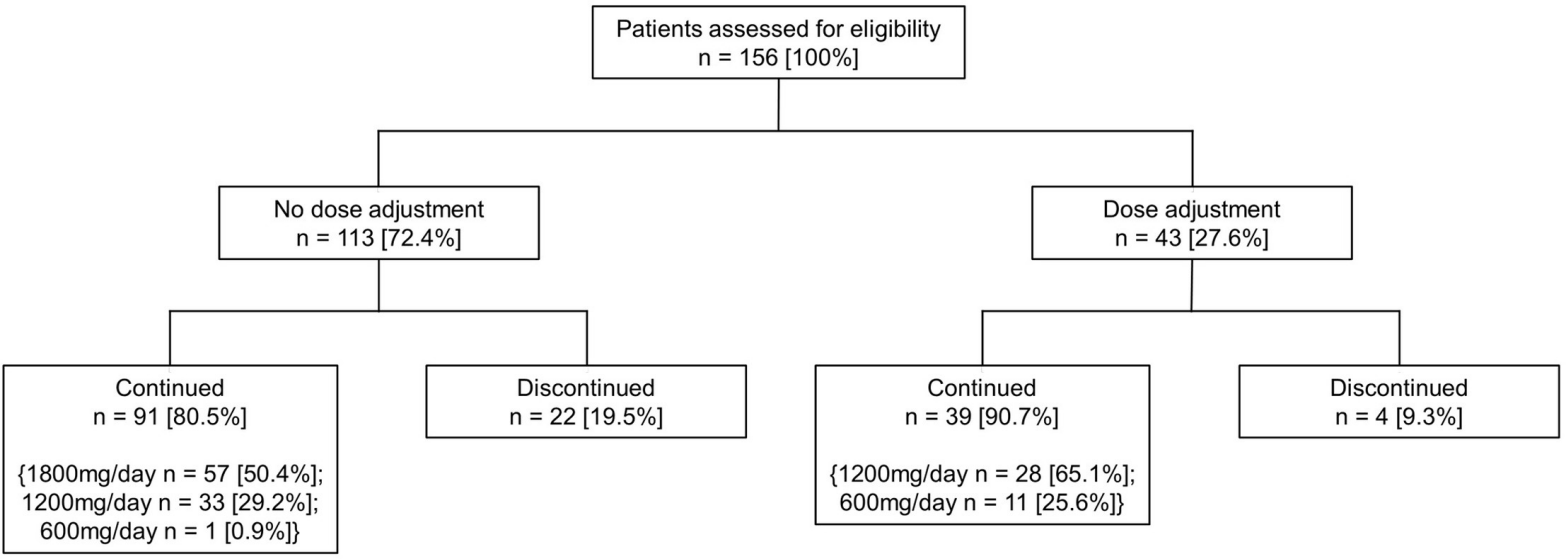
Patient disposition.

This study included 156 patients who received pirfenidone treatment for IPF. The median follow-up period was 243 days (interquartile range [IQR]: 84–385 days). The mean patient age was 69.7 years. A total of 90.4% patients were male, and 85.8% were ever-smokers. The study population classified by pirfenidone dose and maintenance is summarized in Fig 1. A total of 26 (16.7%) patients who discontinued pirfenidone treatment during the follow-up period. And 73 (46.8%) patients who did not receive higher than the initial dose or received a reduced pirfenidone dose were classified into the low-dose group.

The low-dose group (n = 73) included older patients (71.0 years vs. 67.4 years, *p* = 0.016), fewer smokers (80.8% vs. 96.4%, *p* = 0.008), and patients with a lower BMI (24.1 kg/m^2^ vs. 25.7 kg/m^2^, *p* = 0.027) than the standard dose group (n = 57) (Table 1). No significant difference was found in the baseline PFT results and exercise capacity between the two groups. In addition, no significant difference was found in the rate of pulmonary hypertension between the two groups; however, more patients in the standard dose group had emphysema (*p* = 0.048) than in the low-dose group. Data regarding emphysema and pulmonary hypertension in patients who underwent serial PFTs are presented in S1 Table.

**Table 1:** Baseline patient characteristics.

	Total (n = 130)	Standard dose group (n = 57)	Low-dose group (n = 73)	*P* value
Age, years	69.4 ± 8.6	67.4 ± 9.6	71.0 ± 7.4	0.016
Male, n (%)	117 (90.0)	55 (96.5)	62 (86.9)	0.029
Ever-smoker, n (%)	113 (86.9)	54 (96.4)	59 (80.8)	0.008
BMI, kg/m^2^	24.8 ± 4.2	25.7 ± 4.9	24.1 ± 3.4	0.027
Diagnosis				0.182
Biopsy proven, n (%)	19 (14.6)	11 (19.3)	8 (11.0)	
Clinical, n (%)	111 (85.4)	46 (80.7)	65 (89.0)	
Pulmonary function test				
%FVC, %	70.9 ± 15.0	73.0 ± 15.1	69.3 ± 14.6	0.167
%FEV1, %	80.5 ± 15.2	81.2 ± 13.3	80.0 ± 16.5	0.646
%DLCO, %	53.3 ± 18.5	54.3 ± 19.0	52.6 ± 18.3	0.618
6MWT, m	420.2 ± 99.8	428.1 ± 106.5	414.7 ± 95.3	0.491
6MWT, lowest SpO_2_, %	91.4 ± 4.9	91.0 ± 5.5	91.7 ± 4.5	0.467
Comorbidities				
Respiratory				
COPD, n (%)	17 (13.1)	5 (8.8)	12 (16.4)	0.198
Lung cancer, n (%)	29 (22.3)	15 (26.3)	14 (19.2)	0.332
History of chemotherapy, n (%)	14 (10.8)	5 (8.8)	9 (12.3)	0.516
*Pulmonary hypertension, n (%)	13 (10.0)	6 (10.5)	7 (9.6)	0.860
Pulmonary thromboembolism, n (%)	2 (1.5)	1 (1.8)	1 (1.4)	>0.999
Obstructive sleep apnea, n (%)	1 (0.8)	0 (0.0)	1 (1.4)	>0.999
Non-respiratory				
GERD, n (%)	47 (36.2)	23 (40.4)	24 (32.9)	0.379
PPI/H2 inhibitor usage, n (%)	47 (36.2)	23 (40.4)	24 (32.9)	0.379
Hypertension, n (%)	47 (36.2)	20 (35.1)	27 (37.0)	0.823
Diabetes mellitus, n (%)	43 (33.1)	16 (28.1)	27 (37.0)	0.284
Dyslipidemia, n (%)	26 (20.0)	12 (21.1)	14 (19.2)	0.791
Ischemic heart disease, n (%)	28 (21.5)	10 (17.5)	18 (24.7)	0.328
Liver cirrhosis, n (%)	5 (3.8)	3 (5.3)	2 (2.7)	0.653
Chronic kidney disease, n (%)	5 (3.8)	3 (5.3)	2 (2.7)	0.653
Depression, n (%)	3 (2.3)	1 (1.8)	2 (2.7)	>0.999
Anxiety, n (%)	3 (2.3)	0 (0.0)	3 (4.1)	0.256
Cancer, n (%)	34 (26.2)	10 (17.5)	24 (32.9)	0.048
History of chemotherapy, n (%)	10 (7.7)	3 (5.3)	7 (9.6)	0.512
Emphysema, n (%)	58 (44.6)	31 (54.4)	27 (37.0)	0.048
Pirfenidone dosage, n (%)				
1800 mg/day	57 (43.8)	57 (100.0)		
1200 mg/day	61 (46.9)		61 (83.6)	
600 mg/day	12 (9.2)		12 (16.4)	

Data are presented as the mean ± standard deviation or numbers (percentages), unless otherwise indicated.

*Pulmonary hypertension was assessed based on echocardiography.

Abbreviations: 6MWT, 6-minute walk test; BMI, body mass index; DLCO, diffusing capacity for carbon monoxide; FVC, forced vital capacity; FEV_1_, forced expiratory volume in one second; SpO_2_, peripheral oxygen saturation; COPD, chronic obstructive pulmonary disease; GERD, gastroesophageal reflux disease; PPI/H2 inhibitor, proton pump inhibitor/histamine-2 receptor inhibitor.

### Adverse events

Among patients taking pirfenidone, 82 (63.1%) patients experienced adverse events (Table 2). The most frequent adverse events were dyspepsia (20.0%), urticaria (18.5%), anorexia (13.1%), and general weakness (5.4%). The incidence of anorexia (3.5% vs. 20.5%, *p* = 0.004), urticaria (10.5% vs. 24.7%, *p* = 0.039), and general weakness (0.0% vs. 9.6%, *p* = 0.018) was higher in the low-dose group than in the standard dose group. No significant difference was found in the incidence of dyspepsia, nausea, diarrhea, rash, and photosensitivity between the two groups. However, after categorization of patients by symptoms, more gastrointestinal adverse events (*p* < 0.01), skin-related adverse events (*p* = 0.020), and uncategorized adverse events (*p* = 0.023) were found in the low-dose group than in the standard dose group.

**Table 2:** Adverse events in enrolled patients.

Adverse events, n (%)	Total (n = 130)	Standard dose group (n = 57)	Low-dose group (n = 73)	*P* value
Gastrointestinal	49 (37.7)	9 (15.8)	40 (54.8)	<0.001
Dyspepsia	26 (20.0)	7 (12.3)	19 (26.0)	0.052
Anorexia	17 (13.1)	2 (3.5)	15 (20.5)	0.004
Nausea	4 (3.1)	0 (0.0)	4 (5.5)	0.131
Diarrhea	2 (1.5)	0 (0.0)	2 (2.7)	0.504
Skin	31 (23.8)	8 (14.0)	23 (31.5)	0.020
Urticaria	24 (18.5)	6 (10.5)	18 (24.7)	0.039
Rash	4 (3.1)	2 (3.5)	2 (2.7)	>0.999
Photosensitivity	3 (2.3)	0 (0.0)	3 (4.1)	0.256
Others	11 (8.5)	1 (1.8)	10 (13.7)	0.023
General weakness	7 (5.4)	0 (0.0)	7 (9.6)	0.018
AST/ALT elevation	2 (1.5)	0 (0.0)	2 (2.7)	0.504
Weight loss	1 (0.8)	1 (1.8)	0 (0.0)	0.438
Myalgia	1 (0.8)	0 (0.0)	1 (1.4)	>0.999

Abbreviations: AST, aspartate aminotransferase; ALT, alanine aminotransferase.

### Factors associated with dose reduction of pirfenidone

Logistic regression analysis was performed to evaluate the risk factors associated with dose reduction of pirfenidone (Table 3). In the univariate analysis, age (odds ratio [OR] = 1.054, *p* = 0.020), male sex (OR = 0.205, *p* = 0.045), ever-smoking status (OR = 0.156, *p* = 0.017), lower BMI (OR = 0.901, *p* = 0.035), and emphysema (OR = 0.492, *p* = 0.049) were associated with dose reduction of pirfenidone. In the multivariate logistic regression analysis, older age (OR = 1.066, *p* = 0.016) was significantly associated with dose reduction of pirfenidone after adjusting for sex, ever-smoking status, BMI and emphysema.

**Table 3:** Factors associated with dose reduction of pirfenidone.

Parameters	Odds ratio	95% confidence interval	*P* value
Univariate analysis			
Patients			
Age, years	1.054	1.008–1.101	0.020
Male sex	0.205	0.044–0.965	0.045
Ever-smokers	0.156	0.034–0.719	0.017
BMI, kg/m^2^	0.901	0.817–0.993	0.035
FVC, % predicted	0.983	0.960–1.007	0.168
FEV1, % predicted	0.995	0.972–1.018	0.643
DLCO, % predicted	0.995	0.977–1.014	0.615
6MWT, m	0.999	0.995–1.003	0.487
6MWT, %	1.030	0.952–1.113	0.464
*Pulmonary hypertension	0.902	0.285–2.847	0.860
Emphysema	0.492	0.243–0.997	0.049
Multivariate analysis			
Age, years	1.066	1.012–1.122	0.016
Male sex	1.504	0.096–23.494	0.771
Ever-smokers	0.130	0.009–1.934	0.138
BMI, kg/m2	0.914	0.820–1.017	0.100
Emphysema	0.700	0.321–1.528	0.371

*Pulmonary hypertension was assessed based on echocardiography.

Abbreviations: 6MWT, 6-minute walk test; BMI, body mass index; DLCO, diffusing capacity for carbon monoxide; FVC, forced vital capacity; FEV_1_ forced expiratory volume in one second.

### Adverse events in old age and young age groups

In the ROC curve analysis, age was associated with dose reduction of pirfenidone, and the optimal cutoff level was 70 years (C-index = 0.628, *p* = 0.006). We compared the differences between patients aged ≥70 years (old age group) and those aged <70 years (young age group) (Table 4). No significant difference in the sex ratio, smoking history, or BMI was found between the two groups. Among adverse events, anorexia (*p* = 0.042) occurred more frequently in the old age group than in the young age group.

**Table 4:** Comparison of baseline characteristics and adverse events according to age.

	Total (n = 156)	Age ≥ 70 years (n = 84)	Age <70 years (n = 72)	*P* value
Male, n (%)	141 (90.4)	77 (91.7)	64 (88.9)	0.557
Ever-smoker, n (%)	133 (85.8)	72 (86.7)	61 (84.7)	0.719
BMI, kg/m^2^	24.7 ± 4.0	24.4 ± 4.4	25.1 ± 3.5	0.256
Gastrointestinal	60 (38.5)	34 (40.5)	26 (36.1)	0.576
Dyspepsia	31 (19.9)	14 (16.7)	17 (23.6)	0.279
Anorexia	20 (12.8)	15 (17.9)	5 (6.9)	0.042
Nausea	6 (3.8)	5 (6.0)	1 (1.4)	0.218
Diarrhea	3 (1.9)	0 (0.0)	3 (4.2)	0.096
Skin	42 (26.9)	22 (26.2)	20 (27.8)	0.824
Urticaria	31 (19.9)	16 (19.0)	15 (20.8)	0.781
Rash	6 (3.8)	3 (3.6)	3 (4.2)	>0.999
Photosensitivity	5 (3.2)	3 (3.6)	2 (2.8)	>0.999
Others	17 (10.9)	10 (11.9)	7 (9.7)	0.663
General weakness	12 (7.7)	9 (10.7)	3 (4.2)	0.126
AST/ALT elevation	4 (2.6)	1 (1.2)	3 (4.2)	0.336
Azotemia	1 (0.6)	1 (1.2)	0 (0.0)	>0.999
Weight loss	2 (1.3)	1 (1.2)	1 (1.4)	>0.999
Myalgia	1 (0.6)	1 (1.2)	0 (0.0)	>0.999

Results presented as the mean ± standard deviation or number of patients (%), unless otherwise indicated.

Abbreviations: AST, aspartate aminotransferase; ALT, alanine aminotransferase; BMI, body mass index.

### Factors associated with discontinuation of pirfenidone

A total of 26 (16.7%) patients discontinued pirfenidone, and 130 (83.3%) patients continued pirfenidone. No significant differences in baseline characteristics were found between patients who discontinued pirfenidone and those who continued pirfenidone. Considering adverse events, general weakness (*p* = 0.030) was significantly more common in patients who discontinued pirfenidone than in those who continued pirfenidone. After classification of adverse events according to symptoms, uncategorized adverse events (*p* = 0.040) occurred more frequently in patients who discontinued the drug than in patients who continued the drug (Table 5). Logistic regression analysis was performed to evaluate the factors associated with discontinuation of pirfenidone. Skin-related adverse events (*p* = 0.033) and uncategorized adverse events (*p* = 0.025) were significantly associated with discontinuation of pirfenidone after adjusting for ever-smoking status (S2 Table).

**Table 5:** Comparison of baseline characteristics and adverse events between patients who continued and discontinued pirfenidone.

	Total (n = 156)	Continued pirfenidone (n = 130)	Discontinued pirfenidone (n = 26)	*P* value
Age, years	69.7 ± 8.5	69.4 ± 8.6	70.8 ± 8.5	0.449
Male, n (%)	141 (90.4)	117 (90.0)	24 (92.3)	>0.999
Ever-smoker, n (%)	133 (85.8)	113 (85.0)	20 (76.9)	0.213
BMI, kg/m^2^	24.7 ± 4.0	24.8 ± 4.2	24.5 ± 2.9	0.698
Gastrointestinal	60 (38.5)	49 (37.7)	11 (42.3)	0.659
Dyspepsia	31 (19.9)	26 (20.0)	5 (19.2)	0.929
Anorexia	20 (12.8)	17 (13.1)	3 (11.5)	>0.999
Nausea	6 (3.8)	4 (3.1)	2 (7.7)	0.262
Diarrhea	3 (1.9)	2 (1.5)	1 (3.8)	0.424
Skin	42 (26.9)	31 (23.8)	11 (42.3)	0.053
Urticaria	31 (19.9)	24 (18.5)	7 (26.9)	0.324
Rash	6 (3.8)	4 (3.1)	2 (7.7)	0.262
Photosensitivity	5 (3.2)	3 (2.3)	2 (7.7)	0.194
Others	17 (10.9)	11 (8.5)	6 (23.1)	0.040
General weakness	12 (7.7)	7 (5.4)	5 (19.2)	0.030
AST/ALT elevation	4 (2.6)	2 (1.5)	2 (7.7)	0.130
Azotemia	1 (0.6)	0 (0.0)	1 (3.8)	0.167
Weight loss	2 (1.3)	1 (0.8)	1 (3.8)	0.306
Myalgia	1 (0.6)	1 (0.8)	0 (0.0)	>0.999

Data are presented as the mean ± standard deviation or number of patients (%), unless otherwise indicated.

Abbreviations: AST, aspartate aminotransferase; ALT, alanine aminotransferase; BMI, body mass index.

### Efficacy of low-dose pirfenidone in IPF

**Fig 2:**
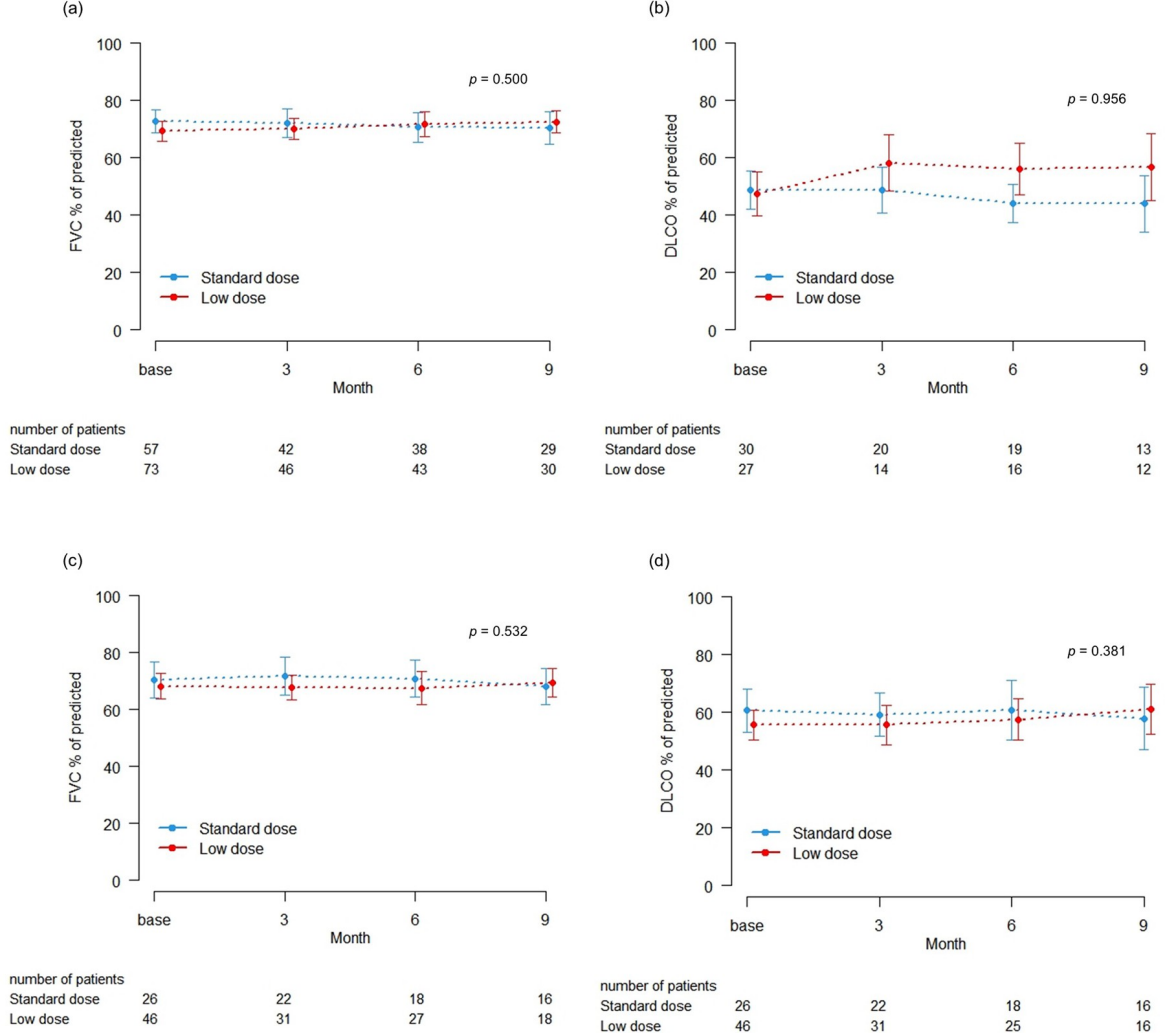
Changes in PFTs according to emphysema and pirfenidone dose. (a) Changes in FVC in patients with emphysema (*p* = 0.500); (b) Changes in DLCO in patients with emphysema (*p* = 0.956); (c) Changes in FVC in patients without emphysema (*p* = 0.532); (d) Changes in DLCO in patients without emphysema (*p* = 0.381). Abbreviations: DLCO, diffusing capacity for carbon monoxide; FVC, forced vital capacity.

To evaluate the efficacy of low-dose pirfenidone on disease progression, we compared the changes in lung function using changes in FVC and DLCO between the standard and low-dose groups, divided by emphysema. Changes in FVC and DLCO after treatment were investigated in patients with data from at least two PFTs at baseline and after treatment. No significant difference was found in the rates of reduced FVC and DLCO between the two groups (Fig 2).

## Discussion

In the present real-world study, 82 (63.1%) patients experienced side effects, and 73 (46.8%) patients did not receive higher doses of pirfenidone up to 1800 mg/day or received a decreased dosage during the follow-up period. Old age was independently associated with dose reduction of pirfenidone; however, no significant difference was found in lung function changes between the standard and low-dose groups.

In previous prospective clinical studies (CAPACITY trials), 98% of patients reported treatment-related adverse events. The most common adverse events were nausea (36%), rash (32%), and dyspepsia (19%) [4]. Similarly, in the pirfenidone use group in the ASCEND trial, 36% of patients experienced nausea, and 28.1% developed a rash. In that study, adverse events occurred more frequently in the pirfenidone use group than in the placebo group, in which 13.4% of patients experienced nausea, and 8.7% developed rash [5]. However, Taniguchi et al. reported that photosensitivity was the most common adverse event in a study population of 267 Japanese patients with IPF (51% of patients in the high-dose group and 53% of those in the low-dose group) [12]. Oltmannus et al. reported that 52 (85%) patients experienced adverse events including fatigue (54%), weight loss (30%), and skin reaction (28%) in real-world practice in Germany [18]. A PMS study in Japan reported that 64.6% of patients experienced side effects [19]. In a single-center study in China, 43.6% of patients with IPF experienced adverse events, which included gastrointestinal (35%) and skin-related (20.5%) adverse events [20]. In the current study, 82 (63.1%) patients experienced adverse events, 49 (37.7%) of whom experienced gastrointestinal adverse events and 31 (23.8%) of whom experienced skin-related adverse events. Hence, there is a possibility that the side effects of pirfenidone may differ between races and may occur at a higher frequency in real world practice than in RCTs.

Based on real-world data, dose reduction of pirfenidone is common [21–25]. Salih et al. reported that of 113 patients with IPF who were treated with pirfenidone, 51 (45.2%) required a dose adjustment, and 18 (16%) discontinued the treatment [21]. In addition, Dbooria et al. reported that of 115 patients with IPF who were treated with pirfenidone, only 49 (42.6%) patients tolerated the full dose (2400 mg/day), and 51 (44.3%) patients tolerated a reduced dose [22]. Although the maximal dose of pirfenidone in Asia is 1800 mg, it has been reported that few patients are actually taking the maximal dose [14, 19]. Indeed, in our study, 73 (46.8%) patients were included in the low-dose group, which is comparable to that reported in previous studies. Although dose adjustment of pirfenidone is known as a method to reduce the occurrence of drug-related adverse events and risk of drug discontinuation [23, 24], few studies have investigated the requirements for dose adjustment and factors associated with dose reduction. However, Uehara et al. suggested that an adjusted dose of pirfenidone for body surface area or body weight could reduce adverse events and promote continued treatment with pirfenidone based on data from a small number of patients [25]. In the current study, old age was independently associated with pirfenidone dose reduction. Considering the findings of previous reports that elderly patients usually experience drug side effects [26], it may be necessary to carefully monitor adverse events, especially in elderly patients with IPF who receive pirfenidone.

In the current study, 26 (16.7%) patients discontinued pirfenidone. The rate of discontinuation of the drug was comparable to that reported in previous studies. In a prospective study by Dhooria et al., 22 (19.1%) patients discontinued the treatment [22]. A study assessing real-world pirfenidone data in Germany reported that 18 (15.4%) patients discontinued pirfenidone. In a study in Greece, nine (20.9%) patients discontinued the treatment [27]. However, the predictors of discontinuation of pirfenidone are not well known. A study reported that low BMI is a predictor of drug discontinuation because of adverse events [22]. Another study reported that older age, use of steroids prior to the study, and female sex were associated with early termination of the treatment [28]. In our study, patients who experienced skin-related or uncategorized adverse events were significantly more likely to discontinue the drug. However, few patients discontinued pirfenidone in our study. Further large-scale studies investigating predictors of discontinuation are warranted.

In a pooled analysis of multinational phase III trials, the efficacy of pirfenidone was compared between patients according to dose intensity (>90% and ≤90%). The study reported that regardless of dose intensity, the pirfenidone group had a significantly lower annual rate of FVC decline than the placebo group [23]. Another phase III trial in Japan reported a significant difference in vital capacity (approximately 0.07 L) between the high-dose (1800 mg/day) and placebo groups (*p* = 0.0416) and a significant difference in vital capacity (approximately 0.09 L) between the low-dose (1200 mg/day) and placebo groups (*p* = 0.0394), suggesting that low-dose pirfenidone is effective [12]. A study assessing real-world pirfenidone data in South Korea reported that relatively a low dose of pirfenidone (≤1200 mg/day) can have an effect on pulmonary function decline [14, 15]. In our study, we compared changes in FVC and DLCO between the standard and low-dose groups and found no significant difference in efficacy with dose, which is comparable to the findings of previous studies. Based on the results of our study, low-dose pirfenidone treatment might be an effective treatment strategy, especially in elderly patients.

This study has several limitations. First, the follow-up period was relatively short. However, in previous studies, the median time to the first adverse event leading to dose modification was 62 days, which is relatively early [11, 29]. Second, because patients visited the hospital intermittently and self-reported adverse events, recall bias may exist. Third, not all patients underwent follow-up PFTs, limiting the evaluation of pirfenidone efficacy. Therefore, the short follow-up period did not significantly affect the evaluation of adverse events; however, it may have affected the evaluation of disease progression.

## Conclusion

The present study revealed that older patients are more likely than younger patients to undergo dose reduction of pirfenidone. Thus, monitoring whether older people experience side effects and show poor compliance is essential. No difference was found in the effect of pirfenidone between the standard and low-dose groups; hence, low-dose pirfenidone could be considered effective, especially for the treatment of older patients with IPF.

## Supporting information

S1 Table Emphysema and pulmonary hypertension in patients who underwent serial PFT. (DOCX)

S2 Table Risk factors for discontinuation of pirfenidone in patients with IPF. (DOCX)
