# Supplementary material for: Factors associated with dose reduction of pirfenidone in patients with idiopathic pulmonary fibrosis: A study based on real-world clinical data
Source: PLoS One. 2023 Feb 3;18(2):e0281295. doi: 10.1371/journal.pone.0281295 (PMC9897553; doi:10.1371/journal.pone.0281295)
Supplement: S1 Table — (DOCX) [file pone.0281295.s001.docx]

|  | **Total**  **(n = 98)** | **Standard dose**  **(n =46)** | **Low dose**  **(n = 52)** | ***P* value** |
| --- | --- | --- | --- | --- |
| Emphysema, n (%) | 42 (42.9) | 24 (52.2) | 18 (34.6) | 0.080 |
| ^*^Pulmonary HTN, n (%) | 10 (10.2) | 5 (10.9) | 5 (9.6) | >0.999 |

**S1 Table. Emphysema and pulmonary hypertension in patients who underwent serial PFTs**

Data are presented as the number of patients (%), unless otherwise indicated.

^*^Pulmonary hypertension was assessed based on echocardiography.

Abbreviations: HTN, hypertension; PFT, pulmonary function test.
