# Supplementary material for: Factors associated with dose reduction of pirfenidone in patients with idiopathic pulmonary fibrosis: A study based on real-world clinical data
Source: PLoS One. 2023 Feb 3;18(2):e0281295. doi: 10.1371/journal.pone.0281295 (PMC9897553; doi:10.1371/journal.pone.0281295)
Supplement: S2 Table — (DOCX) [file pone.0281295.s002.docx]

S2 Table. Risk factors for discontinuation of pirfenidone in patients with IPF

| **Parameters** | **Odds ratio** | **95% confidence interval** | ***P* value** |
| --- | --- | --- | --- |
| Univariate analysis | | | |
| Baseline characteristics |  |  |  |
| Age, years | 1.020 | 0.969-1.074 | 0.449 |
| Male sex | 1.333 | 0.282-6.295 | 0.716 |
| Ever-smokers | 0.472 | 0.165-1.351 | 0.162 |
| BMI, kg/m^2^ | 0.978 | 0.876-1.093 | 0.696 |
| Adverse events |  |  |  |
| Gastrointestinal | 1.212 | 0.516-2.851 | 0.659 |
| ∙∙Dyspepsia | 0.952 | 0.328-2.765 | 0.929 |
| ∙∙Anorexia | 0.867 | 0.235-3.203 | 0.831 |
| ∙∙Nausea | 2.625 | 0.455-15.145 | 0.280 |
| ∙∙Diarrhea | 2.560 | 0.223-29.324 | 0.450 |
| Skin | 2.342 | 0.975-5.626 | 0.057 |
| ∙∙Urticaria | 1.627 | 0.615-4.307 | 0.327 |
| ∙∙Rash | 2.625 | 0.455-15.145 | 0.280 |
| ∙∙Photosensitivity | 3.528 | 0.559-22.249 | 0.180 |
| Others | 3.245 | 1.078-9.767 | 0.036 |
| ∙∙General weakness | 4.184 | 1.214-14.420 | 0.023 |
| ∙∙AST/ALT elevation | 5.333 | 0.716-37.720 | 0.102 |
| ∙∙Weight loss | 5.160 | 0.312-85.254 | 0.252 |
| Multivariate analysis | | | |
| Ever-smoker | 0.382 | 0.127-1.150 | 0.087 |
| Skin-related adverse events | 2.723 | 1.086-6.823 | 0.033 |
| Other adverse events | 3.715 | 1.183-11.663 | 0.025 |

Abbreviations: AST, aspartate aminotransferase; ALT, alanine aminotransferase; BMI, body mass index; IPF, idiopathic pulmonary fibrosis.
